# The Solvent Role for the Decomposition of Paracetamol in Distilled and Drinking Water by Pure and Ag-Modified TiO_2_ Sol–Gel Powders

**DOI:** 10.3390/ma17081791

**Published:** 2024-04-13

**Authors:** Albena Bachvarova-Nedelcheva, Reni Iordanova, Nina Kaneva

**Affiliations:** 1 Institute of General and Inorganic Chemistry, Bulgarian Academy of Sciences, Acad. G. Bonchev Str., bld. 11, 1113 Sofia, Bulgaria; reni@svr.igic.bas.bg; 2 Laboratory of Nanoparticle Science and Technology, Department of General and Inorganic Chemistry, Faculty of Chemistry and Pharmacy, University of Sofia, 1 James Bourchier Blvd., 1164 Sofia, Bulgaria; nina_k@abv.bg

**Keywords:** sol–gel, Ag/TiO_2_ powders, paracetamol, photocatalytic degradation, distilled and drinking water

## Abstract

In this study, pure TiO_2_ gels were synthesized by applying the sol–gel method, using Ti(IV) butoxide with the addition of two different solvents, namely ethylene glycol (EG) and isopropanol (isop), with only air moisture present. It was established using XRD that the gel prepared with the addition of EG was amorphous even at 400 °C, while the other gel was amorphous up to 300 °C. It was found that TiO_2_ (anatase) had a dominant crystalline phase during heating to 600 °C, while at 700 °C, TiO_2_ (rutile) appeared. The as-obtained powdered materials were annealed at 500 °C and subsequently underwent photocatalytic tests with paracetamol. Additionally, the TiO_2_ samples were modified with Ag^+^ co-catalysts (10^−2^ M), using photofixation by UV illumination. The photocatalytic activity of the Ag-modified powders was also tested in the photodegradation of a commonly used paracetamol in aqueous solution under UV light illumination. The obtained data exhibited that the annealed samples had better photocatalytic efficiency and decomposed paracetamol faster in comparison to the non-annealed sol–gel powders. The highest degradation efficiency was observed for the TBT/isop/Ag material, with degradation efficiencies average values of 65.59% and 75.61% paracetamol achieved after the third cycle of photocatalytic treatment. The co-catalytically modified powders had higher photocatalytic efficiency in comparison to the pure nanosized powders. Moreover, the sol–gel powders of TBT/EG, TBT/EG/Ag (10^−2^ M), TBT/isop, and TBT/isop/Ag (10^−2^ M) demonstrated the ability to retain their photocatalytic activity even after three cycles of use, suggesting that they could find practical use in the treatment of pharmaceutical wastewater. The observed photocatalytic efficiency and positive impact of silver make the prepared powders a desirable choice for pharmaceutical drug degradation, helping to promote environmentally friendly and effective wastewater treatment technology.

## 1. Introduction

Nowadays, the sol–gel method is mainly used for the production and synthesis of nanoparticles. It has a higher popularity and industrial application due to its unique properties and characteristics, which allow the production of high-quality nanoparticles of the same size on an industrial scale [1,2]. This method allows for the possibility of obtaining two or more types of nanoparticles simultaneously, meaning that alloy products are synthesized in one step by mixing two or more metal (or metal oxide) precursors in certain proportions [3]. Furthermore, very high-purity, highly homogenous composites can be produced using the sol–gel process [4,5]. Another advantage in comparison with the conventional methods is the lower temperature of the process, which makes the production of metal and ceramic nanomaterial possible at temperatures below 500 °C [6,7,8]. The sol–gel process is a bottom-up synthesis method in which the final products are formed by performing a number of irreversible chemical reactions [9]. During these reactions, the primary homogeneous molecules (sol) become a heavy, three-dimensional molecule called a gel [10]. The narrow particle size distribution, consistent nanostructure at low temperatures, and high product purity are the key benefits of the sol–gel technique. It is commonly used to synthesize metal nano-oxides. As previously stated, the sol–gel process involves changing the state from sol to gel using a variety of techniques, most of which use gentle drying to remove the solvent [1]. One of the most crucial aspects of this method is creating suitable conditions to stop cracks as a result of the gel drying.

Organic compounds that are generated in various industries produce problematic pollutants in water. Pharmaceutical compounds are considered emerging contaminants in aquatic environments that are not easily eliminated by conventional treatment processes. Acetaminophen (paracetamol) is one of the most often used pharmaceuticals, which is used in over a hundred pharmaceutical products [11,12,13,14]. Pollution is a global issue, and recently, the treatment of wastewater has become crucial due to the detrimental effects of contaminants and the danger that contaminated wastewater poses to humans, animals, and agriculture. Physical, biological, and chemical procedures are involved in the treatment of wastewater in order to protect the environment from contamination [15].

Semiconductors using various metal oxides, such as TiO_2_, SnO_2_, CeO_2_, ZrO_2_, WO_3_, and ZnO, have been explored as excellent photocatalysts in order to degrade organic pollutants in wastewater. One of the most promising is TiO_2_, especially for the photocatalytic removal of environmental contaminants, since at room temperature and pressure in water, a number of organic molecules can be broken down into small pieces or go through mineralization. Numerous publications have discussed its efficacy as a photocatalyst for the degradation of various pollutants, such as dyes or phenolic compounds like paracetamol [12]. The literature review revealed that the photodegradation of pollutants, as well as microorganism inactivation, is due to recombination processes. Electron–hole pairs migrate on the TiO_2_ surface and subsequently generate highly reactive oxygen species (ROS) [16]. ROS react with the impurities adsorbed on the surface, leading to their decomposition into harmless compounds [17]. However, the quick transport of electrons and holes [12,15] reduces their potential for photocatalytic degradation [15,18,19,20]. Aiming to improve catalysts’ efficiency, different metal and non-metal additives have been investigated [21,22,23]. Recently, it has been found that Ag/TiO_2_ is a promising photocatalyst, playing an important role in the removal of a large range of pollutants in aqueous solutions under UV and visible light [17]. Silver nanoparticles as antibacterial agents have aroused great interest compared to other metals [24,25]. It has been generalized that the photocatalytic behaviors of Ag/TiO_2_ nanoparticles toward different pollutants depend on several parameters, such as the type of metal precursors; solvents; reducing agents; and other parameters such as pH, temperature, water/precursor molar ratio, etc. [17]. Differences in the photoactivity of silver-doped TiO_2_ prepared with different methods were observed, mostly explained by the oxidation state of silver on the TiO_2_ surface, the particle size that should be in the nanometer scale, silver concentration loading in the final photocatalyst, and thermal treatment during the photocatalyst preparation [17]. Evidently, the understanding of TiO_2_ properties is still stimulating scientific interest.

One of the primary tasks developed by our team is using the sol–gel method to obtain various binary, ternary, and multicomponent composite powders containing TiO_2_. Investigations on the structure, thermal, optical, photocatalytic, and antibacterial properties of selected compositions have been performed [26,27,28,29]. This work expands our research on the sol–gel synthesis of TiO_2_ nanocomposite powders.

The main focus of this paper is to study the influence of two different solvents—ethylene glycol (EG) and isopropanol (isop)—in the synthesis of TiO_2_ using the sol–gel method. The photocatalytic behavior for the degradation of acetaminophen (ACT) was studied as well. The photocatalytic studies were performed by following the degradation of an aqueous solution of paracetamol under the action of ultraviolet light, using the sol–gel powder photocatalysts. Additionally, the as-obtained TiO_2_ powders co-catalytically modified with Ag(I) ions by photo-fixation were also investigated for the degradation of ACT in distilled and drinking water. A comparison of the obtained pure gels with those of Ag/TiO_2_ powders was made, which highlights the paper’s originality.

## 2. Materials and Methods

### 2.1. Preparation of the Gels

The sol–gel processing was performed using the following precursors: Ti(IV) butoxide—TBT (>98%, Merck, Rahway, NJ, USA) for introducing the titania as well as ethylene glycol (EG, C_2_H_6_O_2_, >99.5%, Merck) and isopropanol (i-PrOH, >99.5%, Merck, New Jersey, USA) as dissolvents. Isopropyl alcohol is a fast-evaporating common solvent, reagent, and disinfectant that is used as an industrial cleaning agent. The usage of these solvents in the sol–gel process is preferable as both facilitate the generation of homogeneous gels.

Two gels were prepared using Ti(IV) n-butoxide without the direct addition of water, and the sol–gel hydrolysis reaction was accomplished only in the presence of air moisture. One of the gels was obtained using ethylene glycol (EG) as a dissolvent, while the other one was prepared by dissolving the Ti butoxide in isopropanol (both in a 1:1 molar ratio), denoted on the figures as Ti(IV) but/EG (TBT/EG) and Ti(IV) but/isop (TBT/isop). According to the literature data [30,31], the use of diols as solvents is preferable due to their ability to modify metal alkoxides and act as chelating ligands forming bridges with other alkoxide groups. In this way, the obtained solutions become sufficiently stable. The resulting internal H-bond network was not disrupted as the surfactant was not added [32]. The pH of the as-prepared solutions was measured at 4–5. The aging of TBT/EG and TBT/iPr gels was performed in the air for several days to allow for further hydrolysis. After that, they were subjected to stepwise heating in the air up to 700 °C for one hour of exposure time for each temperature. In the temperature range of 150–200 °C, the bulk samples were broken into small pieces during the drying process. Further increasing temperatures (300–700 °C) was important to verify the phase transformations of the gels.

The silver co-catalytically modified TiO_2_ powders were produced via chemical photodeposition. The sol–gel samples (TBT/EG and TBT/isop) were photofixed for twenty minutes under ultraviolet light using an aqueous AgNO_3_ solution (10^−2^ M), and then the powders were rinsed with water. According to Wegnerich and Mul [33], the irradiation of a titanium dioxide semiconductor and silver precursor in an aqueous solution for a certain period is sufficient to reduce silver ion (Ag^+^) to silver metal (Ag^0^) and ensure their deposition on the TiO_2_ surface. To eliminate NO_3_^–^, the modified samples were dried for ten minutes at 100 °C. In earlier studies, Kaneva et al. [34,35] successfully employed the chemical photodeposition method to break down organic dyes like malachite green and methylene blue as well as photofixed ZnO and TiO_2_ sol–gel films with silver ions. According to experimental and structural properties, a silver co-catalytically modified semiconductor has better properties than pure films. As a result, we were able to produce and examine the photocatalytic characteristics of pure and Ag^+^ co-catalytically modified sol–gel TiO_2_ (TBT/EG and TBT/isop) powders for the degradation of paracetamol dissolved in distilled and drinking water.

The pharmaceutical product ACT (λ_max_ = 243 nm, 99.0%) from Teva was chosen as a model drug for the photocatalytic experiments. In the photocatalytic tests, drinking water and distilled water were used to show how an analgesic degrades in the presence of different impurities, as it does in the environment. In Sofia, Bulgaria, drinking water is not dangerous for daily consumption and helps keep body cells functioning normally, due to the safe concentration of soluble salts (Na^+^ < 5.01 mg/L, Ca^2+^ < 10.74 mg/L, Cl^−^ < 5 mg/L, NO_3_^–^ < 0.94 mg/L, etc.; pH = 7.39). The main sources of drinking water are mountain water sources, which are situated close to the “Beli Iskar” reservoir and are more than 2.5 km above sea level. Although the water from the “Beli Iskar” reservoir is naturally pure and from an upland source, it still needs to meet European law-making standards. It has to go through a purification station first to eliminate organic substances that have solvated microorganisms.

### 2.2. Samples’ Characterization

Powder XRD patterns were registered at room temperature in an air atmosphere. The powder patterns were recorded using a Bruker D8 Advance powder diffractometer (Billerica, MA, USA) with CuKα radiation (k = 1.54056 Å) and a high-speed LynxEye PSD detector (Singapore) within the 2θ range starting from 10 up to 80° with step size 0.02° 2θ and 17.5 s/step in continuous scanning mode. The average crystallite size of anatase nanocrystals was determined with the Scherrer equation D = Kλ/βcosθ, using the full width at half-maximum of the diffraction peaks at 2θ = 25.38°, where D is the average crystallite size in nanometers, λ is the wavelength of the applied X-ray radiation, β is the full width at half-maximum of the diffraction peak, and θ is the diffraction angle. The qualitative phase analysis was performed using the Rietveld method and Topas 4.2 program [36]. All crystal structure parameters of Rutile and Anatase were kept fixed, except for the unit cell and profile parameters (background coefficients, zero point error, FWHM of the Bragg peaks, and the scale factor).

The infrared spectra were registered in the range 1600–400 cm^−1^ using the KBr pellet technique on a Nicolet-320 FTIR spectrometer (Madison, WI, USA) with 64 scans and a resolution of ±1 cm^−1^. The optical absorption spectra of the powdered samples in the wavelength range 200–800 nm were recorded with a UV–visible diffused reflectance spectrophotometer “Evolution 300” (Carson, CA, USA) using a magnesium oxide reflectance standard as the baseline. The band gap energies (*E*_g_) of the samples were calculated using Planck’s equation:

$$E_{g}=\frac{h.c}{\lambda }=\frac{1240}{\lambda }$$

where *E*_g_ is the band gap energy (eV), *h* is Planck’s constant, *c* is the light velocity (m/s), and *λ* is the wavelength (nm).

The as-obtained samples were imaged with a scanning electron microscope (SEM) JSM-5510 (JEOL, Tokyo, Japan), operated at 10 kV of acceleration voltage. The investigated samples were coated with gold using a JFC-1200 fine coater (JEOL) before observation. The specific surface areas (BETs) were determined by low-temperature (77.4 K) nitrogen adsorption in NOVA 127 1200e, Quantachrome, Boynton Beach, FL, USA surface area and pore analyzer at relative pressures p/p_0_ = 0.1–0.3 using the BET equation. The elemental composition was analyzed by energy-dispersive X-ray spectroscopy (EDS) using Bruker AXS detector (Microanalysis GmbH, Berlin, Germany).

The photocatalytic experiments were conducted in a 400 mL glass reactor equipped with a magnetic stirrer rotating at 500 rpm and a 36 W UV lamp (315–400 nm emission range) emitting light between 315 and 400 nm in wavelength. The tests were carried out at 23 ± 2 °C, room temperature. The decomposition of paracetamol dissolved in distilled and drinking water was used to compare the photocatalytic activity of pure and silver co-catalytically modified TiO_2_ powders. The results are the mean values of three measurements of each sample. The analgesic solution had a 250 mL volume. The drug (0.5 g) was dissolved in 0.5 L of water to create the standard main solution. To create usable solutions with 50 ppm, this primary solution was diluted. Following aliquot sampling (0, 1, 2, 3, and 4 h), the pollutant’s mineralization process was assessed using UV–visible absorbance spectroscopy (spectrophotometer Evolution 300 Thermo Scientific (Waltham, MA, USA), wavelength range: 200–600 nm). UV–visible spectra were plotted as KMU (Kubelka–Munk units) vs. λ (wavelength in nm).

Using an Elementar Vario Select TOC analyzer (Langenselbold, Germany) and the 850 °C catalytic oxidation method, the total organic carbon (TOC) of the treated dye solutions was measured. Based on three measurements for every sample, the observed standard deviation was computed. The following formula was used to determine the percentage of TOC removal:

$$Mineralization\;(TOC)\;\%=\frac{{TOC}_{i}-TOC}{{TOC}_{i}}\times 100$$

where TOC_i_ is the initial TOC of the paracetamol, and TOC is the final TOC at time t.

## 3. Results and Discussion

### 3.1. Phase Transformations

**Figure 1:**
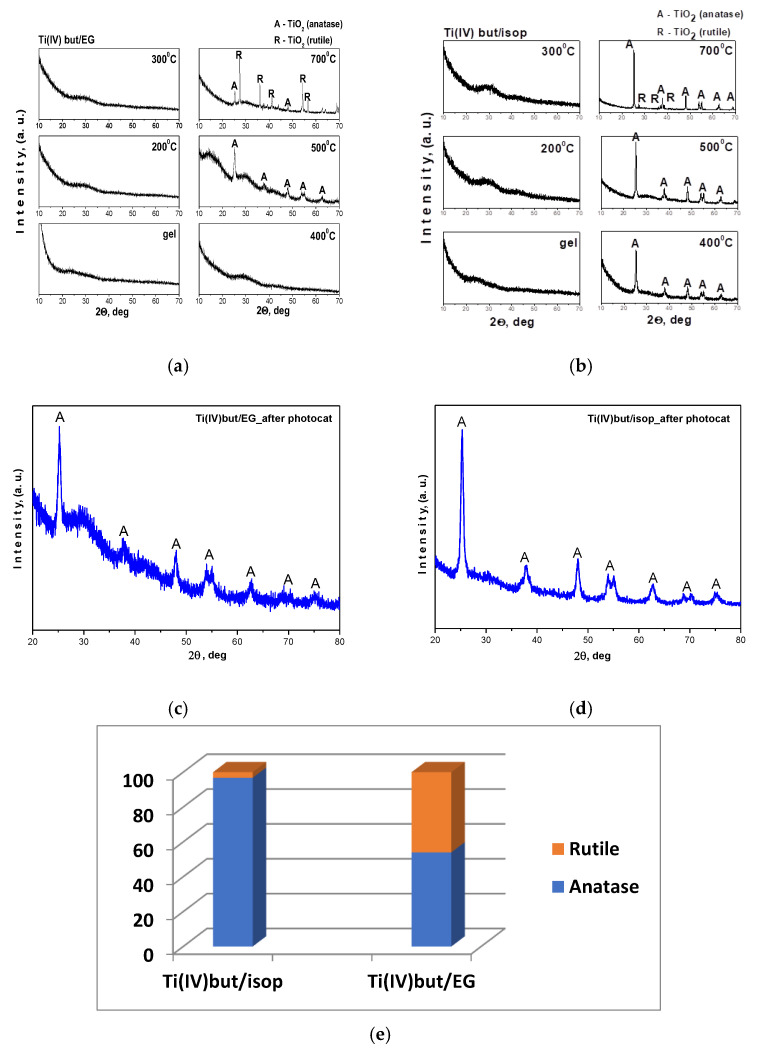
XRD patterns of Ti(IV) n-butoxide dissolved in EG (Ti(IV) but/EG) (**a**); isopropanol (Ti(IV) but/isop) (**b**) heat-treated at different temperatures and after photocatalysis: (A) TiO_2_ anatase, (R) TiO_2_ rutile (**c**,**d**); crystalline phase weight content of the studied samples at 500 °C (**e**).

The XRD patterns of as-prepared gels heat-treated in the temperature range of 200–700 °C are shown in Figure 1a,b. As can be seen, both samples were amorphous up to 300 °C, but the TBT/EG sample also preserved the amorphous state at 400 °C. Further increase in temperature led to the appearance of first TiO_2_ (anatase) crystals (JCPDS 78-2486) and then TiO_2_ (rutile) (JCPDS 21-1276) at 700 °C. The XRD patterns of the sample obtained with the addition of EG showed the presence of an amorphous halo even at 500 °C. Possibly, the presence of organic groups from that dissolvent led to the stronger stabilization of the amorphous state at higher temperatures. On the other hand, there was a dominant-phase TiO_2_ (rutile) at 700 °C in the TBT/EG sample. Obviously, EG as a solvent only in combination with Ti(IV) butoxide led to a more complete transformation of TiO_2_ (anatase) to rutile at higher temperatures. At 500 °C, the average crystallite size (calculated using Sherrer’s equation) of TiO_2_ (anatase) in all samples was about 5–10 nm. At the higher temperature (600 °C), the crystallite size increased, and it was about 15–20 nm. These phase formation results are consistent with previous authors’ reports [37,38]. Most of the authors claimed that, at higher temperatures, the crystallization rate of solid phases increases due to the large movement of atoms, which facilitates the rapid arrangement of the crystalline structure and the subsequent aggregation of the crystallites aiming to minimize the interfacial surface energy [39]. At 700 °C, no presence of an amorphous phase was observed, and only anatase and rutile were detected as crystalline phases, which were quantified (Figure 1e). The unit cell parameters for sample Ti(IV)/isop were determined for TiO_2_ (anatase) as a = 3.7859(3) Å, c = 9.5154(7) Å and for TiO_2_ (rutile) as a = 4.594(2) Å, c = 2.958(3) Å. For the other sample Ti(IV)/EG, the unit cell parameters were as follows: TiO_2_ (anatase)—a = 3.7855(3) Å, c = 9.5162(15) Å; TiO_2_ (rutile)—a = 4.5949(2) Å, c = 2.9605(3) Å.

Figure 1c,d show the XRD patterns of investigated samples after photocatalysis. The results exhibited that the samples after use were still invariant, and they could be effectively used as photocatalysts [40].

### 3.2. SEM Investigations

**Figure 2:**
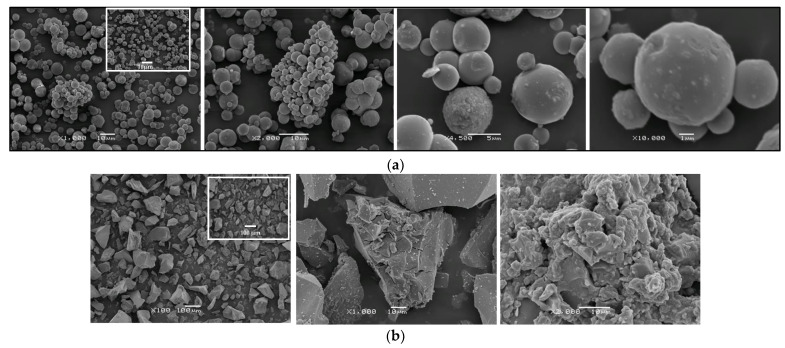
(**a**) SEM images at different magnifications of the Ti(IV) n-butoxide dissolved in isopropanol (Ti(IV) but/isop) heat-treated at 500 °C. (**b**) SEM images at different magnifications of the Ti(IV) n-butoxide dissolved in EG (Ti(IV) but/EG) heat-treated at 500 °C. SEM images of the used catalysts are shown as insert images.

The samples’ surface morphology was examined by SEM (Figure 2). The micrographs at different magnifications represent the obtained powders calcined at 500 °C. The comparison of the images in Figure 2a,b shows that the particle morphology of TBT/EG differed from that of the TBT/isop sample. Figure 2a shows the SEM observations of TBT/isop powders. The micrographs showed well-defined nanoparticles with a spherical shape, which could be related to the solvent isopropanol probably playing an important role during the sol–gel process. The estimated particle size was about 5 µm. The SEM analysis for the TBT/EG pattern (Figure 2b) exhibited TiO_2_ particles with plate-like or irregular shapes, along with a strong trend of agglomeration. The average size of the aggregates was above 10 µm. The micrographs also indicated that the particles obtained with the sol–gel method usually exhibited strong agglomeration and less porous structure due to the lack of surfactant, which could reduce the agglomeration and size of the nanoparticles [41]. Additional confirmation for the agglomeration of the samples is the BET measurements, which showed that the specific surface area of the annealed TBT/EG and TBT/isop powders were 33 and 98 m^2^/g, respectively. The non-annealed sol–gel titanium dioxide nanostructures had lower BET values (TBT/EG 28 m^2^/g and TBT/isop 93 m^2^/g). The larger specific surface of the annealed samples, established by the BET analysis, is one of the prerequisites for their higher photocatalytic activity compared to the non-annealed catalysts. Further, SEM images of the used catalysts are shown as insert images in Figure 2a,b. The surface morphology of as-prepared materials was not changed after the photocatalytic degradation of paracetamol under ultraviolet illumination. This provides evidence of the stability of the catalysts.

**Figure 3:**
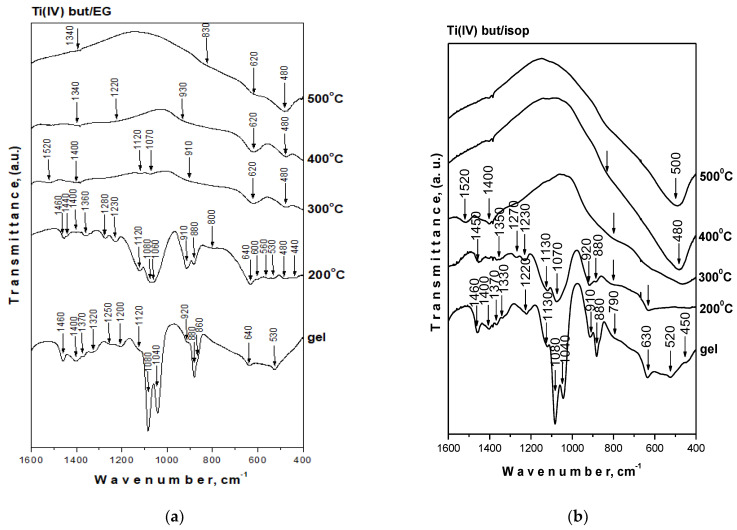
IR spectra of Ti(IV) but/EG and Ti(IV) but/isop heat-treated at different temperatures. Ti(IV) but/EG (**a**) and Ti(IV) but/isop (**b**).

It has already been found that the amount of -OH groups from the dissolvent helps to control the reaction rate, which leads to a smaller particle size [42]. Additionally, several authors [43,44] stated that the molar ratio between TBT and water helps to control the shape of particles. These results confirmed that the estimated crystallite sizes from the XRD and the variation in the size and shape of TiO_2_ particles could be explained in terms of the completeness of the hydrolysis–condensation reactions. These processes were completed to a greater extent in the TBT/isop sample, as discussed below in Section 3.3 “Structural Investigations” (Figure 3). It was mentioned above that no water was added during the sol–gel process. Obviously, the presence of the chosen dissolvent (TBT and isopropanol) was one of the main factors in controlling the shape and size of the particles. These findings correlate well with other investigations [43,44].

Energy-dispersive X-ray spectroscopy (EDS) was used to examine the elemental composition of TBT/EG/Ag and TBT/isop/Ag powders to determine the presence of Ti, O, and Ag chemical elements in the samples with the highest (10^−2^ M) silver content. According to the EDS analysis, the weight percentages of elements in the TBT/EG/AG were Ti 31.92%, O 59.23%, and Ag 8.85%, and in the TBT/isop/Ag sample, these values were Ti 30.8%, O 60.68%, and Ag 8.52%. The reason for this is the small amounts of co-catalysts that were added during photofixation. This outcome shows that it is possible to effectively create Ag co-catalytically modified TiO_2_ nanostructure materials that are photofixed under UV light.

### 3.3. Structural Investigations

Figure 3 displays the infrared spectra of the compositions under investigation heated at various temperatures. Based on spectral data for the precursors and our earlier structural studies on gels in various binary and ternary TiO_2_-containing systems, the vibration band assignments were determined [45,46,47,48]. The completeness of the hydrolysis and condensation processes was also verified by IR spectroscopy. Looking at the spectra, all samples exhibit similar behaviors in the absorption range 900–400 cm^−1^. The bands in this region are related to the C-H, C-O, and Ti-O-C deformation vibrations [49,50]. It is known that Ti(IV) butoxide is characterized by the band at 1130 cm^−1^ typical for the stretching vibrations of Ti-O-C, while those at 1100 and 1040 cm^−1^ are assigned to the vibrations of terminal and bridging C-O bonds in butoxy ligands. The bands located between 1500 and 1300 cm^−1^ are attributed to the bending vibrations of CH_3_ and CH_2_ groups [49,50,51]. Additionally, bands at 1090 and 1040 cm^−1^ are characteristic of ethylene glycol. They are assigned to both symmetric and asymmetric stretching vibrations of C-O bonds in CH_2_-OH groups. Special attention was paid to the band changes at 1120, 1080, and 1040 cm^−1^ in order to evaluate the degree of hydrolysis of the investigated samples (Figure 3). These unhydrolyzed organic groups are responsible for the formation of a mixed organic–inorganic amorphous structure that exists at low temperatures. As shown in the spectra of the gel compositions, the absorption band at 1040 cm^−1^ of the sample TBT/isop significantly decreased in comparison to the TBT/EG one. Obviously, the addition of isopropanol stimulated a higher degree of hydrolysis–condensation reactions in that sample. The wide absorption band between 800 and 400 cm^−1^ could be related to the Ti–O vibration [52]. Increasing the temperatures led to the transformation of the organic–inorganic amorphous network to an inorganic one in which bands of inorganic structural units could only be distinguished in the IR spectra.

### 3.4. UV–Visible Spectroscopy

**Figure 4:**
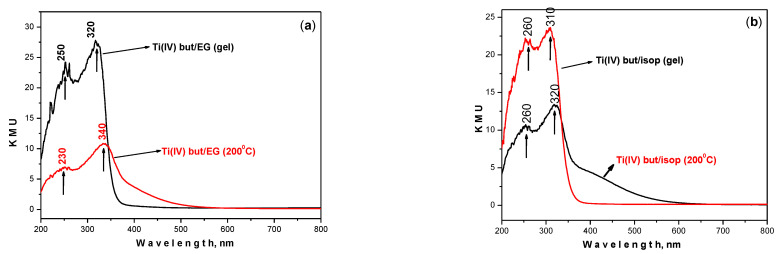
UV–visible spectra of Ti(IV) n-butoxide dissolved in EG (Ti(IV) but/EG) (**a**) and isopropanol (Ti(IV) but/isop) (**b**) gels heat-treated at 200 °C.

UV–visible spectroscopy was used in order to obtain information on the optical behavior of the TiO_2_ powders as well as useful data for the completion of the hydrolysis–condensation reactions. The UV–visible curves of the samples are shown in Figure 4. Generally, the spectra appear similar in shape and position of the bands. The main difference is that the cut-off of Ti(IV) but/isop gel (364.55 nm) (Figure 4b) is red-shifted as compared to the Ti(IV) but/EG gel (350.39 nm) (Table 1). On the other hand, the Ti(IV) but/isop sample heated at 200 °C (Figure 4b) exhibited stronger and sharper bands centered at 260 and 320 nm in comparison to the Ti(IV) but/EG one (200 °C, Figure 4a). Obviously, the type of alcohol used as a solvent affected the optical properties in a different way. These results could be related to the effect of particle size, which is known to influence the UV absorption edge [43]. The determination of the band gap of the nanoparticles is an important parameter for the size effect. The optical band energies of the anatase TiO_2_ nanoparticles, as estimated from the absorption onset, are shown in Table 1. As can be seen, the E_g_ value for TBT/isop is smaller (E_g_ = 3.40 eV) than that of TBT/EG (E_g_ = 3.54 eV). This fact and the sharper absorption peaks in the UV–visible spectra of TBT/isop are additional proof of the change in the crystalline phase and the average particle size [53]. This statement is in accordance with Dakhlaoui et al. [54], who suggested that smaller particles have higher intensities for the UV peak. On the other hand, it is known that the band gap of pure titanium dioxide powder is about 3.2 eV. Looking back to the E_g_ values of the investigated gel samples, it can be seen that they are slightly higher than that of pure TiO_2_. This experimental fact could be related to the presence of organics, which has been stated by other authors [55].

The smaller particles and larger surface area (98 m^2^/g) of the TBT/isop sample make it a promising candidate for photocatalytic applications [53,56]. The occurrence of smaller TiO_2_ NPs was confirmed by the greater absorbance at about 400 nm [57]. The Ti(IV) but/isop (Figure 4b) sample showed sharper and stronger bands centered at 260 and 320 nm, as previously mentioned. Therefore, it could be concluded that the stronger absorption band at 320 nm was related to the higher number of TiO_6_ structural units and consequently more completed hydrolysis–condensation reactions in the Ti(IV) but/isop sample. From these results, it can be summarized that the use of isopropanol resulted in TiO_2_ particles with smaller grain sizes, which influenced the optical properties of the obtained samples. Similar results concerning the impact of the solvent on the optical properties have been reported by Mahyar et al. [58]. Future studies assessing the influence of the solvent and particle size on the band gap and optical properties will be performed.

### 3.5. Photocatalytic Efficiency of Pure and Silver-Photofixed Sol–Gel TiO_2_ Powders

**Figure 5:**
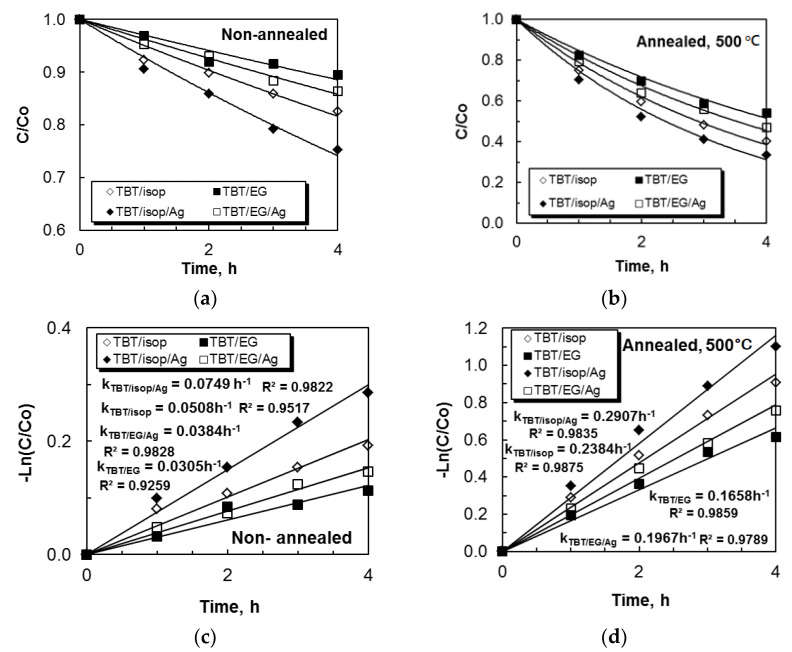
The time course of the decrease in the concentration for the mineralization of paracetamol using non-annealed (**a**) and annealed (**b**) pure and silver-photofixed TiO_2_ sol–gel powders (TBT/isop and TBT/EG). Kinetics of the photocatalytic decomposition of analgesic in distilled water (**c**,**d**) under UV light illumination are illustrated.

Initially, we assessed the photocatalytic properties of non-annealed and annealed TiO_2_ sol–gel (TBT/EG and TBT/isop) powders for the decomposition of paracetamol in distilled water using ultraviolet irradiation. There was no discernible deterioration of the analgesic solution in the control experiment, which involved exposing an aqueous solution of ACT in the absence of a photocatalyst. Figure 5a,b show the concentration profiles of analgesic, which decreased with time in ultraviolet light. The photocatalytic results showed that non-annealed TBT/isop powders had higher activity in comparison with non-annealed TBT/EG. This finding is well explained by a less porous structure, the absence of a surfactant, and the presence of a solvent that affects the shape and size of the particles. The TBT/isop sample’s smaller particles, greater surface area (98 m^2^/g), and lower E_g_ value (E_g_ = 3.40 eV) are preconditions for the faster degradation of paracetamol. Compared to the non-annealed samples, the annealed samples demonstrated a significantly higher degree of photocatalytic efficiency. Considering the surface morphology of the TiO_2_ powders, it can be concluded that the higher degree of crystallinity as well as the porous and rough surface structure are responsible for the increase in photocatalytic activity with an increase in the powder annealing temperature. The degree of crystallinity has been shown to influence photocatalytic properties [59,60]. Another possibility is the growth of the active surfaces during the annealing process. Therefore, the annealed TBT/isop powders degraded paracetamol faster in comparison with TBT/EG. The average values of the percentage degradation of analgesic from the three cycles in distilled water are given in Table 2.

**Figure 6:**
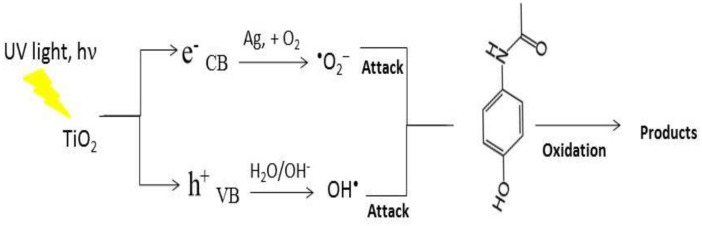
The likely mechanism of paracetamol degradation by pure and silver-photofixed titanium dioxide using ultraviolet illumination.

The second type of photocatalytic test is related to studying the influence of Ag^+^ co-catalytic modification on the titanium dioxide (TBT/isop and TBT/EG) powders in the photocatalytic decolorization of the drug in distilled water. The silver-photofixed samples had higher activity compared to the pure (non-annealed and annealed) semiconductors. This was confirmed by the values of the rate constants (Figure 5c,d). The rate constant, k, is represented by the slope of the linear fits on a logarithmic scale. All of the correlation coefficients (R^2^) were higher than 0.9. For the four TiO_2_ powders, the effectiveness of catalysts was significantly improved with the photofixed silver ions, and the rate constant (*k*) increased. This was observed in both non-annealed (from 0.0508 to 0.0749 h^−1^ for TBT/isop, from 0.0305 to 0.0384 h^−1^ TBT/EG; Figure 5c) and annealed (from 0.2384 to 0.2907 h^−1^ for TBT/isop, from 0.1658 to 0.1967 h^−1^ for TBT/EG; Figure 5d) powders. Evidently, in our photocatalytic tests, the presence of silver was helpful in the degradation of the drug under UV irradiation. Higher values of the k and degree of degradation (Table 2) indicate faster ACT elimination because ultraviolet light can excite e^-^ in the catalyst and oxidize the organic pollutant. This result could also be explained by the narrowing of the band gap width in the silver-containing samples and a decrease in the rate of the recombination of the generated e^−^/h^+^ pairs [61]. The schematic representation of the possible mechanism for the degradation of analgesic using pure and silver-photofixed titanium dioxide powders is given in Figure 6.

TiO_2_ absorbs light, which excites an electron to move from the valance band (VB) to the conduction band (CB), creating a hole in the valance band and causing a charge separation. Through Schottky heterojunction at the TiO_2_/Ag interface, the photogenerated electrons located in the CB of TiO_2_ are transferred to Ag [62]. Through their reaction with dissolved oxygen, superoxide radicals are created [63]. Hydroxyl radicals are concurrently produced by photoinduced holes in the TiO_2_ VB reacting with H_2_O or OH- attached to the surface. For the photocatalytic process to degrade organic pollutants, the generated superoxide and hydroxyl radicals are extremely potent oxidants. Ag-photofixed TiO_2_ may have higher photocatalytic degradation efficiency because it improves photoinduced charge carrier separation and delays photoinduced electron–hole pair recombination [64], serving as an electron trap, capturing electrons that move from the TiO_2_ semiconductor’s conduction band and transferring them to oxygen, whereupon they are transformed into superoxide radicals. Hydroxyl radicals are created when water molecules react with photogenerated holes in the valence band, which are still present on TiO_2_. Therefore, the photocatalytic degradation of paracetamol dissolved in distilled and drinking water is an effective method using these free radicals.

**Figure 7:**
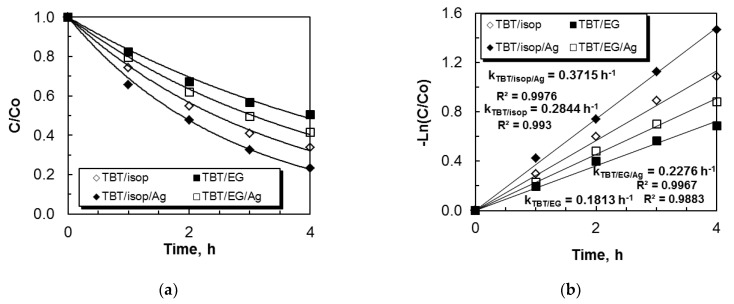
Decrease in the paracetamol concentration in drinking water versus time of UV light illumination with non-annealed and annealed pure and silver-photofixed TiO_2_ powders (**a**,**b**).

The third type of photocatalytic experiment we conducted is related to the degradation of paracetamol in drinking water. Ag/TBTisop powders exhibited the highest photocatalytic efficiency (k = 0.3715 h^−1^), as shown by Figure 7a,b, which illustrates the same trend in photocatalytic procedures in drinking water conducted in the presence of UV illumination. The activity of the TBT/EG modified with silver was higher (k = 0.2276 h^−1^) than that of the pure sample. Comparing the paracetamol degradation rate in distilled water to that in drinking water, the pure and Ag-modified nanostructure samples had a lower rate. The differences in pH levels are responsible for this observation. pH controls photocatalysis, as is widely known. The pH values of distilled water (pH = 6.5) and drinking water (pH = 7.39) differ from each other, albeit not significantly. The drinking water in which paracetamol was dissolved had a slightly higher pH than distilled water. This means that drinking water had a higher concentration of hydroxyl ions, which led to the formation of more hydroxyl radicals in the valence band. Therefore, a higher number of free radicals is the reason for the faster and more efficient degradation of paracetamol in drinking water compared to when it is dissolved in distilled water. Using Ag-ZnO catalysts, Ramasamy et al. [65] conducted a more thorough investigation into the effects of pH on the degradation of paracetamol. They found that the rate of drug degradation increased as the pH increased from 4 to 8.5. This may be explained by electrostatic forces existing between the contaminants and the catalyst. At pH values greater than the point of zero charge, the catalyst surface is negatively charged, and vice versa. The acid-ionization constant of paracetamol is 9.5. Consequently, an increase in pH above 9 damages elimination and decreases the rate constant by progressively accelerating the electrostatic attraction between contaminants and the modified surface semiconductor. Our results related to silver-modified titanium dioxide powders are consistent with previous studies investigating ZnO/Ag catalysts [65,66,67].

**Figure 8:**
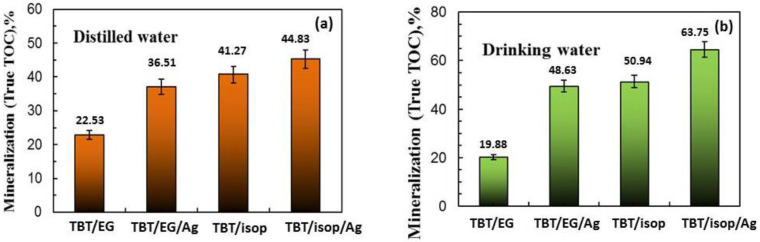
TOC analysis, measured at the end of the third cycle of photocatalytic reaction in (**a**) distilled water and (**b**) drinking water.

The photocatalytic data were confirmed by total organic carbon values. TOC measurements were carried out in order to examine the degradation of paracetamol in both water after 4 h of ultraviolet light illumination, as shown in Figure 8. The TOC percentages for the mineralization of analgesic were found in distilled (TBT/EG 22.53% and TBT/isop 41.27%) and drinking (TBT/EG 19.88% and TBT/isop 50.94%) water, respectively.

The TOC conversion rates of analgesic photodegradation increased when silver ions (10^−2^ M) were co-catalytically modified into semiconductor TiO_2_ powders (distilled water: TBT/EG/Ag—36.51% and TBT/isop/Ag—44.83%; drinking water: TBT/EG/Ag—48.63% and TBT/isop/Ag—63.75%), but the trend was the same. However, it was discovered that the TOC removal percentages of paracetamol in drinking and distilled water were marginally lower than the rate at which they broke down during photocatalytic processes, which was caused by the development of intermediate by-products.

The decomposition of paracetamol was found to have lower values with TOC analysis than with UV–visible spectroscopy, demonstrating that the process involved multiple complex steps and intermediate products. For instance, several intermediate products from the photocatalytic oxidation of analgesic were identified [68]. When interacting with OH^-^ radicals generated during the photocatalytic process, each of these by-products has a distinct oxidation potential.

**Figure 9:**
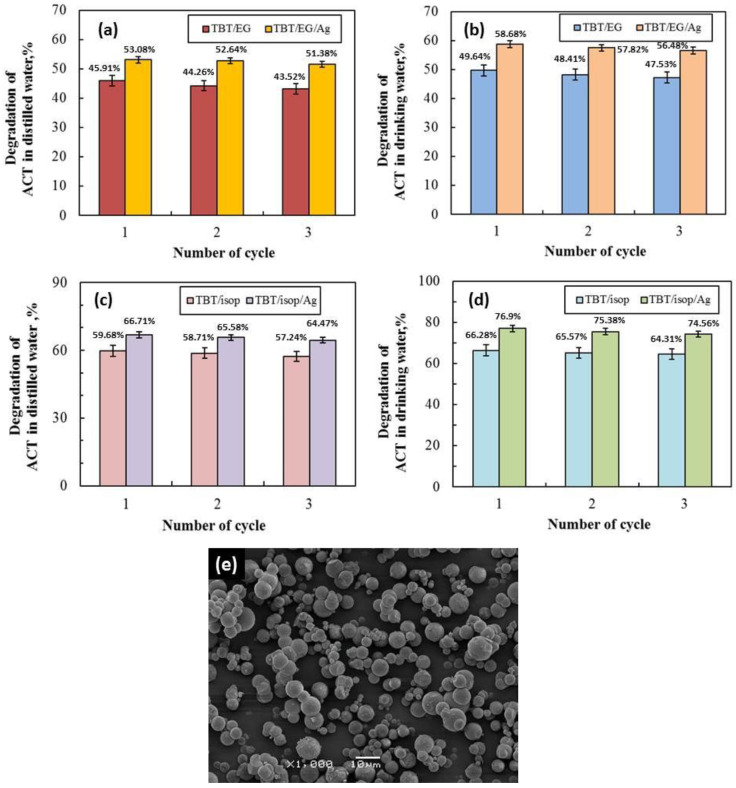
Photostability of TBT/EG and TBT/EG/Ag (**a**,**b**) and TBT/isop and TBT/isop/Ag (**c**,**d**) sol–gel powders on the decomposition of paracetamol in distilled and drinking water using UV illumination; surface morphology and integrity of the starting material (**e**).

Heterogeneous photocatalysis is an environmentally friendly technology that usually has no problems with waste disposal. As a result, maintaining high photocatalytic efficiency is crucial for the duration of each usage cycle. Figure 9 presents the results of an investigation into the regeneration and repurposing of TBT/EG, TBT/EG/Ag (10^−2^ M), TBT/isop, and TBT/isop/Ag (10^−2^ M) sol–gel powders. It is evident that the catalytic properties of the powders gradually decreased with each cycle. After three cycles, the drug’s photocatalytic degradation decreased by roughly 2% for the four kinds of catalysts in distilled and drinking water. After three cyclical experiments, the results verified that TBT/isop/Ag (10^−2^ M) co-catalytically modified nanostructures were still highly photocatalytically active. The powder’s SEM image (Figure 9e) shows that the surface morphology and integrity of the starting material are preserved.

## 4. Conclusions

With the inclusion of two distinct solvents (ethylene glycol and isopropanol) and only air moisture, pure titania gels were made from Ti(IV) butoxide. It was verified that these solvents are appropriate for producing homogenous and transparent gels. The presence of ethylene glycol preserved the mixed organic–inorganic amorphous structure at higher temperatures (400 °C), while in the other sample at this temperature, the first TiO_2_ (anatase) crystals appeared. The phase transition of TiO_2_ (anatase) to TiO_2_ (rutile) was registered above 600 °C. The type of the dissolvent influenced the shape, size, and properties of the powders.

Silver ions (10^−2^ M) were modified into TiO_2_ sol–gel powders by photofixation under UV light. The photocatalytic decomposition of paracetamol was faithfully portrayed by the pseudo-first-order kinetic model. The photocatalytic data show that the elimination of paracetamol in drinking and distilled water under UV light is enhanced by the addition of silver ions to TiO_2_. Due to the reduction in photogenerated electron–hole pair recombination and electron transfer from titanium dioxide’s conduction band to Ag, the metal’s oxidizing power improved. The TBT/isop/Ag powders had the highest percentages of paracetamol degradation under UV light exposure (Ddistilled = 66.7%, Ddrinking = 76.9%) in both distilled and drinking water. The system’s improved photocatalytic efficiency and the advantageous effects of silver ions render it a desirable choice for the breakdown of pharmaceuticals, thereby promoting the development of environmentally sustainable and effective wastewater treatment methods.

## Figures and Tables

**Table 1:** Cut-off and optical band gap values (E_g_) of the investigated powders.

Sample	Cut-off, nm		E_g_, eV	
	Gel	200 °C	Gel	200 °C
Ti(IV)/isop	365.55	353.43	3.40	3.50
Ti(IV)/EG	350.39	382.57	3.54	3.24

**Table 2:** Degree of paracetamol mineralization in distilled water under 240 min ultraviolet light.

Samples	Average Values of Degradation of Paracetamol in Distilled Water, %
TBT/EG	10.62
TBT/EG/Ag	13.63
TBT/isop	17.50
TBT/isop/Ag	24.82
TBT/EG, 500C	44.56
TBT/EG/Ag, 500C	52.37
TBT/isop, 500C	58.54
TBT/isop/Ag, 500C	65.59

## Data Availability

Data are contained within the article.
